# Evaluation of Th1/Th2, regulatory cytokines and transcriptional factor FoxP3 in sheep immunized with a partially protective and non-protective vaccine and challenged with *Fasciola hepatica*

**DOI:** 10.1186/s13567-024-01308-8

**Published:** 2024-04-24

**Authors:** María Teresa Ruiz-Campillo, Isabel Lourdes Pacheco, Nieves Abril, María José Bautista, Álvaro Martínez-Moreno, Francisco Javier Martínez-Moreno, Leandro Buffoni, José Pérez, Verónica Molina-Hernández, Rafael Zafra

**Affiliations:** 1 Departamento de Anatomía y Anatomía Patológica Comparadas y Toxicología, Facultad de Veterinaria, UIC Zoonosis y Enfermedades Emergentes ENZOEM, Universidad de Córdoba, Edificio de Sanidad Animal, Campus de Rabanales, Ctra. Madrid‐Cádiz Km 396, 14014 Córdoba, Spain; 2 Departamento de Bioquímica y Biología Molecular, Universidad de Córdoba, Edificio Severo Ochoa, Campus de Rabanales, Ctra. Madrid‐Cádiz Km 396, 14014 Córdoba, Spain; 3 Departamento de Sanidad Animal (Parasitología), Facultad de Veterinaria, UIC Zoonosis y Enfermedades Emergentes ENZOEM, Universidad de Córdoba, Edificio de Sanidad Animal, Campus de Rabanales, Ctra. Madrid‐Cádiz Km 396, 14014 Córdoba, Spain

**Keywords:** *Fasciola hepatica*, cytokines, FoxP3, sheep, vaccine, liver, hepatic lymph nodes

## Abstract

Gene expression for Th1/Th2 cytokines (IL-4 and IFN-ɣ), regulatory cytokines (TGF**-**β and IL**-**10) and the transcriptional factor FoxP3 was analyzed in the liver and hepatic lymph nodes (HLN) from sheep immunized with partially protective and non-protective vaccine candidates and challenged with *Fasciola hepatica*. FoxP3 T cells were also evaluated by immunohistochemistry (IHQ). The most remarkable difference between the partially protected vaccinated (V1) group and the non-protected vaccinated (V2) group was a more severe expansion of FoxP3 T cells recorded by IHQ in both the liver and HLN of the V2 group as compared to the V1 group, whereas no differences were found between the V2 group and the infected control (IC) group. Similar results were recorded for FoxP3 gene expression although significant differences among V1 and V2 groups were only significant in the HLN, while FoxP3 gene expression was very similar in the V2 and IC groups both in the liver and HLN. No significant differences for the remaining cytokines were recorded between the V1 and V2 groups, but in the liver the V2 group shows significant increases of IFN-ɣ and IL-10 as compared to the uninfected control (UC) group whereas the V1 group did not. The lower expansion of FoxP3 T cells and lower increase of IFN-ɣ and IL-10 in the partially protected vaccinated group may be related with lower hepatic lesions and fluke burdens recorded in this group as compared to the other two infected groups. The most relevant change in regulatory cytokine gene expression was the significant increase of TGF-β in the liver of IC, V1 and V2 groups as compared to the UC group, which could be related to hepatic lesions.

## Introduction

The helminth parasite *Fasciola hepatica* is the causative agent of fasciolosis, affecting a wide range of mammals but particularly sheep and cattle producing high economic losses to the livestock industry worldwide [1]. The disease has been recognized as an important zoonosis in Africa, Asia, Europa, America and Oceania due to the ingestion of freshwater wild plants [2]. Anthelmintic drugs have been used to control the disease but development of resistance and the presence of drug metabolites in meat and milk [3] have triggered a major interest in the development of an immunological method to control the disease [4]. The parasite *F. hepatica* uses different mechanisms to modulate the host immune response ensuring its survival and making the host immune response ineffective in killing the parasite. This modulation is a serious obstacle in creating protective vaccines for ruminants [5]. FoxP3 regulatory T cells have been considered to play a key role in modulation of immune responses during infections as well as the expression of regulatory cytokines TGF**-**β and IL**-**10. During early stages of *F. hepatica* infection in sheep, the expansion of FoxP3 coincides with an increase in IL-10 gene expression [6], a finding also reported in *Teladorsagia circumcincta* chronic infections in sheep [7]. Recently, studies found the expression of FoxP3 in HLN and liver of goats and sheep in early stages of the disease [6, 8].

On the contrary, some studies have evaluated the downregulation of Th1 response by *F. hepatica* and the mechanism by which the parasite polarizes the immune response to a non**-**protective Th2 response in early stages in sheep [9] and chronic stages in cattle [10–12]. The imbalance towards a Th2 immune profile is mediated through regulatory cytokines [13]. In rats, chronic *F. hepatica* infections induce mixed Th1/Th2/Treg responses [14].

Despite important international efforts none of the vaccine candidates against *Fasciola* spp. has reached a commercial or precommercial stage of development, however, several recombinant candidates have shown promising results in cattle and sheep trials. Thus, the recombinant cathepsin L1 (CL1) reduced fluke burden by 48% in cattle [15] and recombinant leucine aminopeptidase-LAP reduced fluke burden by 74–86% in sheep when using AdjuVac^®^ and alum as the adjuvants, respectively [16]. More recently, a cocktail of four recombinant antigens from *F. hepatica* (CL1, LAP, peroxiredoxin-Prx and helminth defense molecules-HDM) induced a 44% fluke burden reduction in sheep using Montanide^®^ ISA 61 VG as the adjuvant, but it did not induce protection using alum as the adjuvant [17]. A 67% fluke reduction in sheep vaccinated with phage particles encoding a CL1 peptide was reported [18]. Deep knowledge of mechanisms of the immune response in animals receiving protective vaccines versus infected controls and in animals receiving non-protective vaccines are required for further development of efficient vaccine candidates. The aim of the present study was to analyze the gene expression of Th1/Th2 cytokines (IFN-ɣ and IL-4), regulatory cytokines (TGF**-**β and IL**-**10) and the transcriptional factor FoxP3 in liver and hepatic lymph nodes from sheep immunized with the protective and non-protective vaccine candidates in infected and non-infected sheep.

## Materials and methods

### Experimental design

The experimental design is described in detail by [17]. Briefly, thirty-one 8-month-old female Merino sheep were obtained from a liver fluke-free farm and maintained in the experimental farm at the University of Cordoba for six months. Animals were tested monthly to confirm the absence of *F. hepatica* infection. The experiment was approved by the Bioethics Committee of the University of Cordoba (approval number 2015PI//038) and General Directorate for Agricultural and Livestock Production of the Andalucian Government (approval number 01/02/2016/012) conducted in accordance with European (2010/63/UE, Decision 2020/569/UE) and Spanish (L32/2007 and RD 1386/2018) directives on animal experimentation.

Thirty-one sheep were distributed into four groups: the Vaccine 1 (V1) group (*n* = 9) was composed of animals immunized subcutaneously with two doses, 4 weeks apart, of a vaccine made with 100 μg of each of the 4 different recombinant antigens from *F. hepatica*: cathepsin L1 (CL1), helminth defense molecules (HDM), peroxiredoxin (Prx) and leucine aminopeptidase (LAP) in 2 mL excipient (1.2 mL of adjuvant Montanide^®^ ISA 61 VG and 0.8 mL sterilized PBS). Four weeks later booster animals were orally infected with 150 metacercaries (mc) in a single dose. The Vaccine 2 (V2) group (*n* = 9) was composed of animals immunized subcutaneously with two doses, 4 weeks apart, of a vaccine made with 100 μg of each of the same four different antigens plus 2 mL of Aluminum (Alhydrogel^®^ adjuvant 2%, Invivogen, CA, USA) and infected with 150 mc in a single dose. The IC Group (*n* = 9) was orally infected with 150 mc in a single dose, in parallel to the V1 and V2 groups, and used as the infected control. The UC group (*n* = 4) was the uninfected control group. Animals were sedated after 16 weeks after infection by intravenous injection 0.75 mL of xylazine (Xilagesic, Calier Laboratories, Spain) and sacrificed by intravenous injection of 6 mL of T61^®^ (Intervet, Spain) containing embutramide, mebezonium iodide and tetracaine hydrochloride.

### Immunohistochemical analysis

In order to assess FoxP3 expression in the liver and HLN tissues, an immunohistochemical study was carried out using the avidin–biotin-peroxidase method described before [6, 8]. Briefly, slides were incubated in citric acid (pH 6.0) followed by heating in an autoclave for 10 min at 121 °C for antigen retrieval. An incubation with 0.3% hydrogen peroxide (Panreac, Barcelona, Spain) in PBS-Tween 80 was done to block endogenous peroxidase activity. Then, the samples were incubated with 25% normal goat serum (Vector Laboratories, Burlingame, California, USA) for 1 h at room temperature and after that an anti-mouse/rat FoxP3 monoclonal antibody-mAb (clone FJK-16 s, rat IgG2a, eBioscience Inc. San Diego, CA, USA) was diluted 1:100 in PBS containing 10% normal goat serum and applied to the slides overnight as the primary antibody. An anti-rat immunoglobulin serum (Vector Laboratories, Burlingame, California, USA) diluted 1:100 was applied for 30 min. Later an avidin–biotin-peroxidase complex (Vector Laboratories, Burlingame, California, USA) diluted 1:50 was applied for 1 h. Tissue sections were then washed three times in Tris-buffered-saline (TBS, pH 7.2) and incubated with the vector NovaRED^®^ peroxidase substrate kit (Vector Laboratories, Burlingame, California, USA) for 5 min. Finally, samples were rinsed in tap water, lightly counterstained with Mayer hematoxylin and mounted with Eukitt^®^ (Freiburg, Germany).

The number of FoxP3 + cells were carried out using three liver slides per animal and five random microphotographs at 200X magnification. It was necessary to develop specific macros to calibrate the appropriate immunostaining intensity and cell size in the biomedical software Image J v.1.51d. The results are expressed as mean ± SD.

### RNA extraction and cDNA synthesis

Sample collection and cDNA synthesis were carried out as described before [6, 8, 20]. Concisely, after washing samples collected from the left liver lobe and HLN in diethylpyrocarbonate (DEPC) biomolecular water, it was immediately snap frozen in liquid nitrogen, individually disrupted in liquid nitrogen, and finally stored at −80 °C. Then samples were homogenized in 1.5 mL of TRIzol^®^ reagent (Ambion Life Technologies, Carlsbad, CA, USA) using a sterilized IKA^®^T10 basic disperser and then cleaned with the RNeasy^®^ Mini Kit (Qiagen, Hilden, Germany) according to the manufacturer’s guidelines. An incubation with RNase-free DNase I (Qiagen, Hilden, Germany) for 15 min was included. Isolated total RNA was finally incubated at 65 °C for 10 min and kept at −80 °C until use. A Nanodrop 2000 spectrophotometer was used to determine the concentration and purity of RNA. In order to determine the RNA integrity number (RIN), an Agilent 2100 Bioanalyzer (Agilent Technologies, Santa Clara, CA, USA) was used. RIN has values ranging from 0 for degraded RNA to 10 for intact RNA [21]. The quality criteria to use RNA samples in the qRT-PCR experiment were the following: (1) RIN values ≥ 8.5; (2) ratios 260/280 about 2; and (3) absence of gDNA. The absence of a gDNA contaminant in RNA samples was evaluated by conducting a PCR reaction directly with the RNA sample, without retrotranscription, and an intraexonic primer pair. Under these conditions, amplification was obtained only from samples in which gDNA was present. The iScript™ cDNA Synthesis Kit (BioRad, Hercules, CA, USA) was used to generate cDNA from 1 μg of total RNA from each sample individually.

### Primer design and absolute quantification of cytokine expression by real-time PCR

The primer pairs for gene expression analysis of the targets IL-4, IFN-ɣ, IL-10, TGF-β and FoxP3 in the liver and HLN tissue were previously designed [6, 9, 22] with Oligo 7 software (Colorado Springs, USA) over specific sequences obtained from the GenBank database. However, primers devoid of hairpin and duplex structure were required to have a high temperature (≥ 68–70 °C) and optimal 3'-∆G (≤  −6 kcal/mol) values for use in two-step 94/68 °C PCR reactions. All PCR products were further verified by nucleotide sequencing. The products (500–800 bp) of several amplification reactions per gene, of a cDNA synthesized from pooled RNA, were mixed and analyzed by gel electrophoresis. The desired (and unique) DNA band was excised from the gel, and the excised product was purified (Wizard^®^ SV Gel and PCR Clean-Up System Protocol, Promega) and sequenced (ABI PRISM^®^ 310, Applied Biosystems). The identity of the sequences was confirmed using the tBLASTx algorithm on the BLAST server at the NCBI databank. The accession numbers of the gene sequences used to obtain the primer sequences and to assess the amplification specificity are indicated in Table 1.

**Table 1:** **Sequences of the primers used to quantify specific ovine genes by real-time PCR**

Genes	Sequences	Amplicon size (bp)	Accession number
IL-4	F 5′-CATGTGCTTGAACAAATTCCTGGGCGGAC-3′ R 5′-TAGCCTTTCCAAGAGGTCTCTCAGCGTAC-3′	124	NM_001009313.2
IFN-γ	F 5′-ACCGATTTCAACTACTCCGGCCTAACTC-3′ R 5′-CAGAAAAACCCAAAAGCACACAGAGCAG-3′	97	NM_001009803.1
IL-10	F 5′-TCAGCCGTGCTCTGTTGCCTGGTCTTCC-3′ R 5′- GGACGTCCCGCAGCATGTGGGGCAG-3′	124	NM_001009327.1
TGF-β	F 5′- GGGCTTTCGCCTCAGTGCCCACTGTTC-3′ R 5′- CAGAGGGGTGGCCATGAGGAGCAGG-3′	151	NM_001009400.1
FoxP3	F 5′-GCCCATCTGGCTGGGAAGATGGCCCAAACC-3′ R 5′- AGAGGTGCCTCCGCACGGCAAACAGG-3′	166	NM_001144947.1

The SsoAdvanced^™^ Universal SYBR^®^ Green Supmermix (BioRad, Hercules, CA, USA) Kit was used in real-time PCR reactions according to the manufacturer’s guidelines. Three technical replicates using 50 ng of cDNA from each animal and 0.3 µM of each primer in a MyiQ^™^2 Two Color Real-Time PCR Detection System (BioRad, Hercules, CA, USA) were used. Cycling conditions consisted of 2 min at 95 °C for Platinum Taq activation followed by 40 two-step cycles for melting (15 s, 95 °C), and annealing/extension (30 s, 70 °C). After 40 cycles, a melting curve analysis was performed (60‒95 °C) to verify the specificity of amplicons. The amplification for all targets was with the same optimal PCR efficiency (100%) and high linearity (r > 0.99) in the range of 20 to 2 × 10^5^ pg of retrotranscribed total RNA input. To guarantee the quality of the retro-transcription and to detect and remove inter-run variation, an inter-run calibrator (IRC) RNA sample, with a known number of transcripts of the A170 gene was introduced in each experiment and reverse transcribed along with the sheep samples. An absolute calibration curve was generated with an in vitro transcribed RNA, conveniently retrotranscribed, that contained a known number of copies as previously described [23, 24]. The number of transcript molecules corresponding to each experimental gene was calculated from the linear regression of the calibration curve.

### Statistical analysis

For immunohistochemical studies and gene expression the results were expressed as mean ± standard deviation (SD) and mean ± standard error of the mean (SEM), respectively. The Kolmogorov–Smirnov test was applied to evaluate if data were normally distributed. Data were analyzed with the Kruskal–Wallis test. *P* values < 0.001 (a) were considered extremely significant, *P* values < 0.01 (b) very significant and *P* values < 0.05 (c) were considered statistically significant. The statistics software GraphPad Prism 7.0 (GraphPad Software, Inc., San Diego, CA, USA) was used.

## Results

### Fluke burdens and hepatic lesions

The results of the vaccine trial in groups V1 and V2, in terms of fluke burden and gross hepatic lesions, were described previously [17]. Fluke burden of (*P* = 0.002) 37.2% was significantly lower in the V1 group than the IC group. However, no differences were found between the V2 and the IC group [17]. Gross hepatic lesions have also been reported previously and they were significantly lower in group V1 than in group IC [17], without significant differences between V2 and IC groups. Histopathological hepatic lesions were also significantly less severe in group V1 than in group IC, without significant differences between V2 and IC groups [19]. These results suggest that vaccine 1 (V1 group) was partially protective in terms of fluke burdens and hepatic lesions [17].

### Immunohistochemistry (IHC)

**Figure 1:**
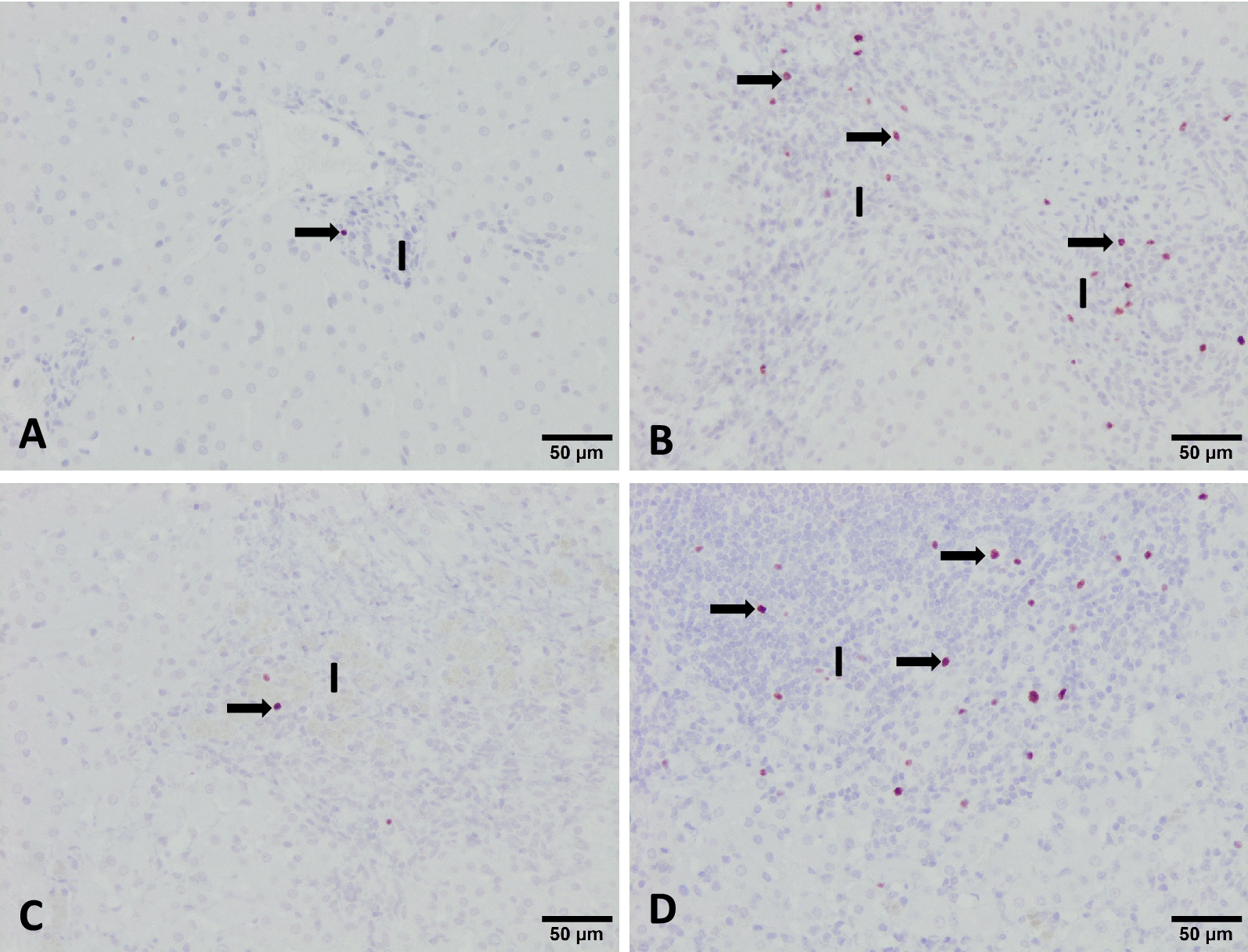
**Photomicrographs of the liver showing FoxP3 + cells (arrows) in the inflammatory infiltrates (I) of portal spaces in the uninfected control (A), infected control (B), vaccine 1 (C) and vaccine 2 (D) groups.** ABC method, haematoxylin counterstain.

**Figure 2:**
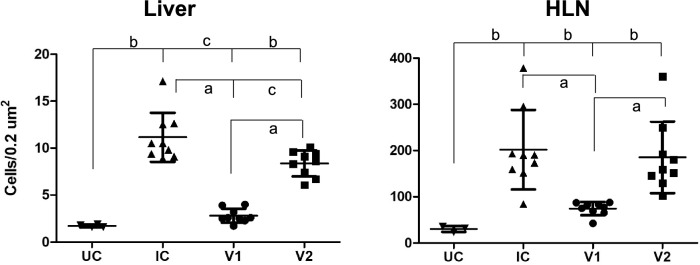
**Quantification of FoxP3 + cells in the liver and hepatic lymph nodes of uninfected control (UC), infected control (IC) and vaccinated groups (V1 and V2) of sheep challenged with** ***F. hepatica*.** Data is represented as mean ± standard deviation (SD). Statistical significance is indicated as **A** *P* values < 0.001, **B** *P* values < 0.01 and **C** *P* values < 0.05.

In the liver, the anti-FoxP3 mAb yielded a nuclear and cytoplasmic immunostaining in lymphocytes mainly located in portal areas (Figure 1). The results of the immunohistochemical study of FoxP3 either in the liver or HLN are summarized in Figure 2. In the liver, FoxP3 + cells in the infected groups (IC, V1 and V2) increased significantly as compared to the UC group (Figure 2). Among the three infected groups, the V1 group shows lower expression of FoxP3 + cells (2.8 ± 0.8) whereas in the IC and V2 it was 11.2 ± 2.6 and 8.4 ± 1.4, respectively with significant differences between the V1 and IC and V1 and V2 groups (*P* < 0.001). In the HLN, the increase of FoxP3 + cells in the three infected groups was significant compared to the UC control, and again this increase was more pronounced in the IC and V2 groups than in V1 (Figure 2).

### Cytokine gene expression

#### Liver

**Figure 3:**
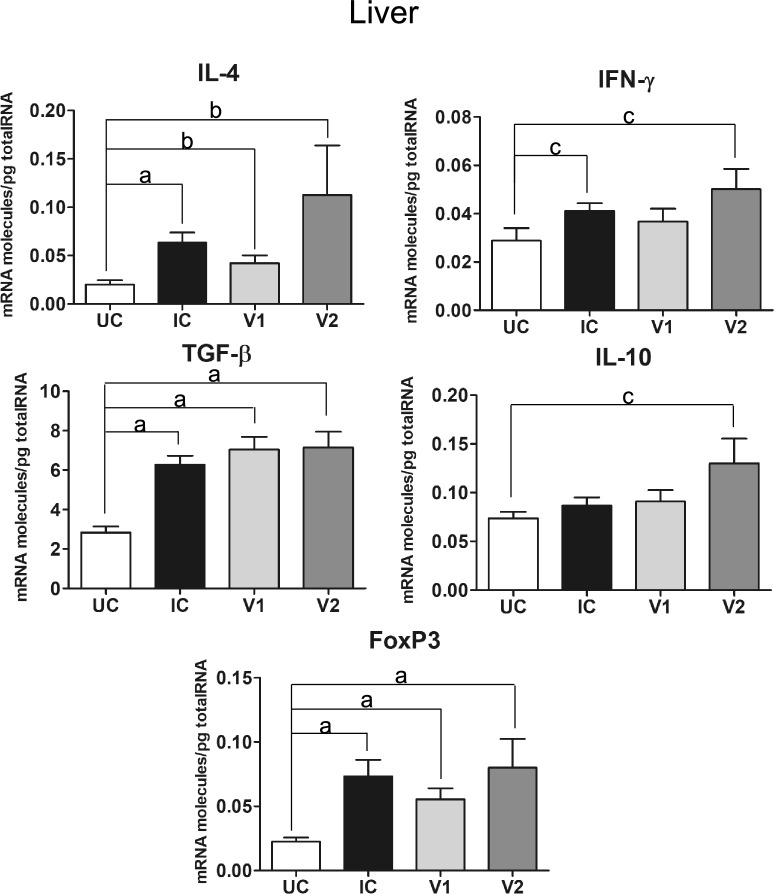
**Gene expression levels in the liver of Th1/Th2 cytokines (IFN-ɣ and IL-4), regulatory cytokines (TGF-β and IL-10) and the transcriptional factor FoxP3 of uninfected control (UC), infected control (IC) and vaccinated groups (V1 and V2) of sheep challenged with** ***F. hepatica***. Each bar represents the mean ± SEM of the mRNA molecules/pg of total RNA quantified individually in each of the animals per group after three real-time PCR reactions per individual. Statistical significance is indicated as **A** *P* values < 0.001, **B** *P* values < 0.01 and **C** *P* values < 0.05.

Gene expression levels in the liver for IL-4 and IFN-ɣ, TGF-β, IL-10 and FoxP3 are shown in Figure 3 and Table 2. Gene expression for IL-4 shows a significant increase in the IC group (*P* < 0.001), and in groups V1 (*P* < 0.01) and V2 (*P* < 0.01) as compared to the UC group (Figure 3). No significant differences were recorded among the three infected groups. Gene expression for IFN-ɣ significantly increased (*P* < 0.05) in the IC and V2 groups as compared to the UC group, whereas the V1 group shows a non-significant increase of IFN-ɣ gene expression as compared to the UC group and a non-significant lower expression as compared to the V2 and IC groups (Figure 3). Expression of TGF-β shows a significant increase (*P* < 0.001) as compared to the UC group, whereas no significant differences were recorded among the three infected groups (Figure 3). Gene expression for IL-10 shows a significant rise (*P* < 0.05) in the V2 group as compared to the UC group, without significant differences among the three infected groups (Figure 3). FoxP3 gene expression shows a significant increase (*P* < 0.001) in the three infected groups as compared to the UC group (Figure 3) without significant differences among the three infected groups.

**Table 2:** **Cytokine gene expression expressed as mean ± SEM of the mRNA molecules/pg of total RNA in the liver and hepatic lymph nodes (HLN) of uninfected control (UC), infected control (IC), vaccine 1 (V1) and vaccine 2 (V2)**

	Liver					
Group	IL-4	IFN-ɣ	TGF-β	IL-10	FoxP3	IFN-ɣ/IL-4
UC	0.02 ± 0.01	0.03 ± 0.01	2.83 ± 0.61	0.07 ± 0.01	0.02 ± 0.01	1.85 ± 1.47
IC	0.06 ± 0.03	0.04 ± 0.01	6.28 ± 1.38	0.09 ± 0.03	0.07 ± 0.04	0.80 ± 0.41
V1	0.04 ± 0.02	0.04 ± 0.02	7.05 ± 1.92	0.09 ± 0.04	0.06 ± 0.02	1.21 ± 1.10
V2	0.11 ± 0.12	0.05 ± 0.02	7.16 ± 2.11	0.13 ± 0.07	0.08 ± 0.06	0.90 ± 0.46

	HLN					
UC	0.44 ± 0.12	0.38 ± 0.15	33.09 ± 2.18	0.63 ± 0.32	1.09 ± 0.17	0.95 ± 0.56
IC	0.61 ± 0.12	0.26 ± 0.15	33.68 ± 9.10	0.63 ± 0.13	1.23 ± 0.55	0.42 ± 0.19
V1	0.47 ± 0.18	0.16 ± 0.08	32.20 ± 3.44	0.55 ± 0.08	0.86 ± 0.20	0.35 ± 0.42
V2	0.55 ± 0.13	0.16 ± 0.03	37.18 ± 8.27	0.49 ± 0.14	1.28 ± 0.29	0.31 ± 0.11

#### Hepatic lymph nodes

**Figure 4:**
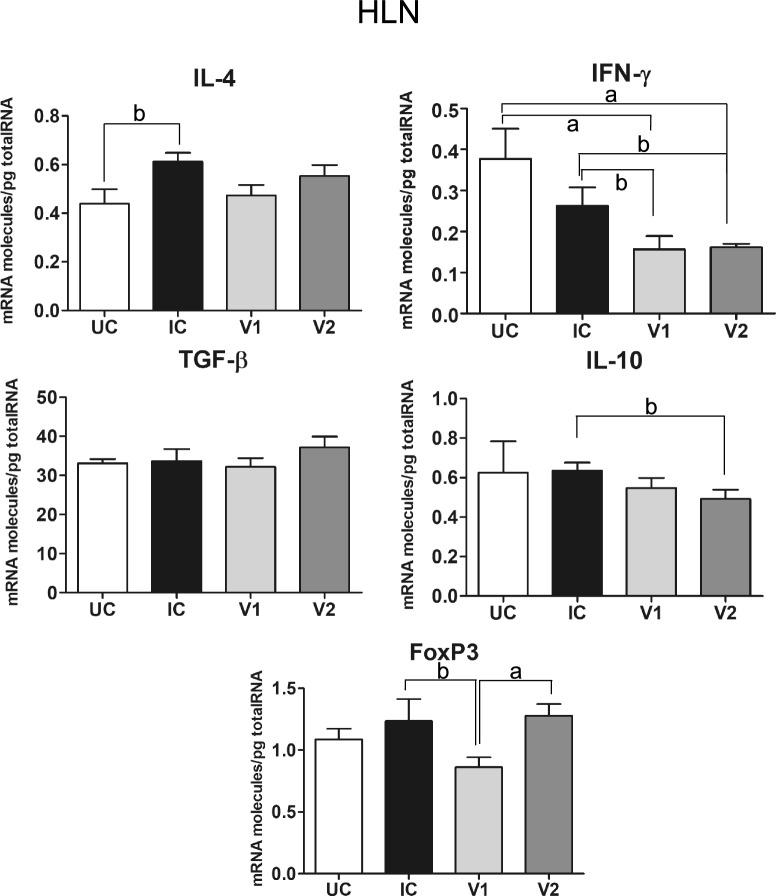
**Gene expression levels in hepatic lymph nodes of Th1/Th2 cytokines (IFN-ɣ and IL-4), regulatory cytokines (TGF-β and IL-10) and the transcriptional factor FoxP3 of uninfected control (UC), infected control (IC) and vaccinated groups (V1 and V2) of sheep challenged with** ***F. hepatica*.** Each bar represents the mean ± SEM of the mRNA molecules/pg of total RNA quantified individually in each of the animals per group after three real-time PCR reactions per individual. Statistical significance is indicated as **A** *P* values < 0.001, **B** *P* values < 0.01 and **C** *P* values < 0.05.

Gene expression levels in HLN for IL-4 and IFN-ɣ, TGF-β, IL-10 and FoxP3 are shown in Figure 4 and Table 2. Gene expression for IL-4 shows a significant increase (*P* < 0.01) in the IC group as compared to the UC group, while no significant differences were recorded in the V1 and V2 groups as compared to the UC and UI groups (Figure 4). IFN-ɣ gene expression in the HLN shows a significant decrease in the V1 and V2 groups as compared to the UC group (*P* < 0.001) and the IC group (*P* < 0.01). No significant differences were recorded between the IC and UC groups and between the V1 and V2 groups (Figure 4). TFG- β gene expression shows no significant differences among the four groups (Figure 4). IL-10 gene expression shows a significant decrease in the V2 group as compared to the IC group, without significant differences among the remaining groups (Figure 4). Finally, the expression of FoxP3 in HLN was significantly higher in the IC (*P* < 0.01) and the V2 group (*P* < 0.001) as compared to the V1group (Figure 4). The V1 group shows a moderate decrease of Foxp3 gene expression compared to the UC group although no significant differences were recorded. Similarly, there were no significant differences for Foxp3 gene expression among the V2, IC and UC groups (Figure 4).

## Discussion

The expansion of FoxP3 + cells detected in the liver both by gene expression and by immunohistochemistry in the three infected groups as compared to the UC group agreed with the expansion of this cell type reported in *F. hepatica* infected sheep [8, 9, 20, 22] and goats [8] as well as in lymph nodes of *F. hepatica* infected cattle [25] and in other helminth infections [7]. In *F. hepatica* infected sheep and goats, this expansion of FoxP3 + T cells has been related with parasite survival and tissue repair [7, 22], which agrees with the results of the present study since in the V1 group the lower FoxP3 + cell expansion coincides with a lower fluke burden and hepatic damage as compared to the other infected groups.

The increase of gene expression for INF-ɣ in the liver of the IC and V2 groups as compared to the UC groups agrees with the increase of this cytokine in the liver of buffaloes chronically infected with *F. gigantica* [26] and the liver of *F. hepatica* infected sheep during early stages of infection [20, 22], but it contrasts with the lack of significant gene expression increase in the liver of chronically *F. hepatica* infected sheep [22] and with the significant decrease of IFN-ɣ expression in the liver of cattle with chronic natural fasciolosis [27]. Splenocytes from mice protected with a Kunitz vaccine also showed increased IFN-ɣ expression [28]. The increased INF-ɣ liver expression during early stages of *F. hepatica* infection in sheep has been attributed to a response to hepatic injuries caused by the parasite such as necrosis and granuloma formation [9]. This hypothesis matches with the results of the present study in which the V1 group shows no significant differences in liver IFN-ɣ gene expression as compared to the UC, whereas the V2 and IC, both with higher hepatic lesions than the V1 group, presented a significant increase of liver IFN-ɣ gene expression as compared with the UC group. By contrast, the decreased IFN-ɣ gene expression in HLN in the present study agreed with the results reported in HLN in previous studies in *F. hepatica* infected sheep [9, 22] and in PBMC of *F. hepatica* infected sheep [29] and cattle [30] and it suggests a downregulation of the Th1 response that may facilitate parasite survival.

In the liver, the three infected groups show significant increases in IL-4 gene expression, whereas in the HLN IL-4 the increase was only significant in the IC group as compared to the UC group. These results matched the increase of IL-4 gene expression during early stages of *F. hepatica* infection in sheep [9, 22] as well as with late stages of *F. hepatica* infection in sheep [18, 22, 31] and cattle [12]. Moreover, reduced serum production of IL-4 was recorded in vaccinated and partially protected sheep using a CL1 phage vaccine prototype [18] which agrees with the lower but not significant reduction of IL-4 gene expression in the partially protected V1group as compared to the non-protected vaccinated group (V2) and infected control group (IC) of the present study.

The decreased IFN-ɣ/IL-4 ratio in the liver and HLN of the infected groups (UC, V1 and V2) with respect to the UC group suggests that *F. hepatica* infection induces a Th2 polarization, which agrees with the results in the liver and HLN [22] and in the serum [18] of chronically *F. hepatica* infected sheep. In the liver, Th2 polarization was less severe in the partially protected vaccinated group (V1) as compared to the non-protected vaccinated group (V2) and the UC group, a finding also reported in partially protected vaccinated sheep serum [18], supporting the hypothesis that a Th2 polarization of the host immune response facilitates parasite survival in *F. hepatica* infections [32].

The increase of TGF-β gene expression in the liver of the three infected groups as compared to the UC group matched with previous results reported in the liver of primarily and secondarily infected sheep at late stages of infection [22] and it contrasted with the absence of increase of this cytokine in the liver of buffaloes chronically infected with *F. gigantica* [27]. However, in the HLN, TGF-β gene expression was not increased in the three infected groups as compared to the UC group, a finding also reported in primarily infected sheep at chronic stages of infection [22], supporting the fact that the expression pattern of this cytokine was different in the liver and HLN during chronic *F. hepatica* infection. TGF-β plays an important role in fibrogenesis [33] which may be related with the increased gene expression in the liver of chronically infected sheep in which hepatic fibrosis is a common lesion [9, 34], whereas in the HLN fibrosis an increase of TGF-β does not occur.

The similar gene expression for IL-10 in the liver of UC and IC groups in the present study agreed with the expression of this cytokine in the liver of sheep chronically infected with *F. hepatica* [22], but it contrasted with the higher levels of IL-10 expression in the livers of buffaloes infected with *F. gigantica* [27], suggesting that the different expression of this cytokine may be due either to the different host species which has been described previously in PBMC of sheep and cattle infected with *F. hepatica* [35] or parasite species. In the livers of sheep during acute stages of *F. hepatica* infection (9 and 18 dpi), a marked increase of IL-10 gene expression was recorded in infected and vaccinated groups with respect to the uninfected control group [9] which suggests a different expression pattern of IL-10 during the migratory and biliary stages of the infection. In the HLN, the absence of differential IL-10 expression among the UC group and the three infected groups agreed with the results of a previous study in sheep primoinfected with *F. hepatica* but contrasted with the increased IL-10 expression in reinfected sheep [22], and also contrasted with the higher expression of this cytokine in the HLN of buffaloes infected with *F. gigantica* during both acute and chronic states of infection [36].

Summarizing, the partially protected vaccinated group (V1) shows a lower FoxP3 T cell expansion in the liver and HLN than the infected control group (IC) and non-protected vaccine group (V2) which may be related with the lower fluke burdens and hepatic lesions in the V1 group. The V2 group shows significant increases in IFN-ɣ and IL-10 as compared to the uninfected control (UC) group in the liver, which was not observed in the V1 group. The lower expansion of FoxP3 T cells and lower increases of IFN-ɣ and IL-10 in the partially protected vaccinated group may be related with the lower hepatic lesions and fluke burdens recorded in this group as compared to the other two infected groups.

## Data Availability

The data presented in this study are available on request from the corresponding author.

## Abbreviations

*F. hepatica*: *Fasciola hepatica*
FoxP3: fork head box P3 transcription factor
IL-10: interleukin 10
TGF-β: transforming growth factor beta
IL-4: interleukin 4
IFN-ɣ: interferon gamma
HLN: hepatic lymph nodes
RNA: ribonucleic acid
cDNA: complementary deoxyribonucleic acid
Th1: T helper type 1
Th2: T helper type 2
Treg: regulatory T cells
qRT-PCR: real time polymerase chain reaction
DEPC: diethylpyrocarbonate
bp: base pair
RIN: RNA integrity number
IHC: immunohistochemistry
UC: uninfected control
IC: infected control
V1: vaccine 1 group
V2: vaccine 2 group
